# The CARTS study: Chemoradiation therapy for rectal cancer in the distal rectum followed by organ-sparing transanal endoscopic microsurgery

**DOI:** 10.1186/1471-2482-11-34

**Published:** 2011-12-15

**Authors:** Guus MJ Bökkerink, Eelco JR de Graaf, Cornelis JA Punt, Iris D Nagtegaal, Heidi Rütten, Joost JME Nuyttens, Esther van Meerten, Pascal G Doornebosch, Pieter J Tanis, Eric J Derksen, Roy S Dwarkasing, Corrie AM Marijnen, Annemieke Cats, Rob AEM Tollenaar, Ignace HJT de Hingh, Harm JT Rutten, George P van der Schelling, Albert J ten Tije, Jeroen WA Leijtens, Guido Lammering, Geerard L Beets, Theo J Aufenacker, Apollo Pronk, Eric R Manusama, Christiaan Hoff, Andreas JA Bremers, Cornelelis Verhoef, Johannes HW de Wilt

**Affiliations:** 1 Department of Surgery, Radboud University Nijmegen Medical Centre, Nijmegen, The Netherlands; 2 Department of Surgery, IJsselland Hospital, Capelle aan de IJssel, The Netherlands; 3 Department of Medical Oncology, Radboud University Nijmegen Medical Centre, The Netherlands; 4 Department of Pathology, Radboud University Nijmegen Medical Centre, Nijmegen, The Netherlands; 5 Department of Radiation Oncology, Radboud University Nijmegen Medical Centre, The Netherlands; 6 Department of Radiation Oncology, Erasmus Medical Centre, Rotterdam, The Netherlands; 7 Department of Medical Oncology, Erasmus Medical Centre, Rotterdam, The Netherlands; 8 Department of Surgery, Academic Medical Centre at the University of Amsterdam, Amsterdam, The Netherlands; 9 Department of Surgery, Slotervaart Hospital, Amsterdam, The Netherlands; 10 Department of Radiology, Erasmus Medical Centre, Rotterdam, The Netherlands; 11 Department of Radiation Oncology, Leiden University Medical Centre, Leiden, The Netherlands; 12 Department of Gastroenterology, The Netherlands Cancer Institute, Antoni van Leeuwenhoek Hospital, Amsterdam, The Netherlands; 13 Department of Surgery, Leiden University Medical Centre, Leiden, The Netherlands; 14 Department of Surgery, Catharina Hospital, Eindhoven, The Netherlands; 15 Department of Surgery, Amphia Hospital, Breda, The Netherlands; 16 Department of Medical Oncology, Amphia Hospital, Breda, The Netherlands; 17 Department of Surgery, Laurentius Hospital, Roermond, The Netherlands; 18 Department of Radiation Oncology, Maastro Clinic, Maastricht, The Netherlands; 19 Department of Surgery, Maastricht University Medical Centre, Maastricht, The Netherlands; 20 Department of Surgery, Rijstate Hospital, Arnhem, The Netherlands; 21 Department of Surgery, Diakonnessenhuis, Utrecht, The Netherlands; 22 Department of Surgery, Medical Centre Leeuwarden, The Netherlands; 23 Division of Surgical Oncology, Erasmus MC, Daniel den Hoed Cancer Center, Rotterdam, The Netherlands

## Abstract

**Background:**

The CARTS study is a multicenter feasibility study, investigating the role of rectum saving surgery for distal rectal cancer.

**Methods/Design:**

Patients with a clinical T1-3 N0 M0 rectal adenocarcinoma below 10 cm from the anal verge will receive neoadjuvant chemoradiation therapy (25 fractions of 2 Gy with concurrent capecitabine). Transanal Endoscopic Microsurgery (TEM) will be performed 8 - 10 weeks after the end of the preoperative treatment depending on the clinical response.

Primary objective is to determine the number of patients with a (near) complete pathological response after chemoradiation therapy and TEM. Secondary objectives are the local recurrence rate and quality of life after this combined therapeutic modality. A three-step analysis will be performed after 20, 33 and 55 patients to ensure the feasibility of this treatment protocol.

**Discussion:**

The CARTS-study is one of the first prospective multicentre trials to investigate the role of a rectum saving treatment modality using chemoradiation therapy and local excision. The CARTS study is registered at clinicaltrials.gov (NCT01273051)

## Background

Colorectal cancer is the third most common malignancy in the Netherlands with more than 10.000 new patients of whom approximately one third have rectal cancer.

Total Mesorectal Excision (TME) using sharp nerve-sparing dissection, instead of blunt resection, reduced the 5-year local recurrence rate from up to 45% to less than 10% in patients with rectal cancer [1,2]. The use of this nerve-sparing technique results in lower rates of sexual dysfunction and urinary incontinence, but these complications are still common after TME [3]. Addition of preoperative radiotherapy to surgery resulted in a significant better local control for resectable rectal cancer. Five-year local control rate using a short course (5 × 5 Gy) of pre-operative radiotherapy was 6% compared to 11% after TME surgery alone [4]. Based on these results, standard treatment in the Netherlands for T2-3 rectal cancer without threatened circumferential margin (CRM) or N2 stage is pre-operative short course radiotherapy followed by TME surgery. Long course chemoradiation therapy (CRT) is indicated for locally advanced disease.

In the majority of patients with rectal cancer a low anterior resection (LAR) with primary anastomosis will be performed. These patients have a risk of significant postoperative morbidity, such as anastomotic leakage, faecal (urge) incontinence, tenesmus and soiling. For patients with distal rectal cancer it is often impossible to preserve the sphincter and an abdominoperineal resection (APR) with a permanent colostomy must be performed. Quality of life in these patients was not proven to be worse than in patients who underwent a LAR [5].

### Neoadjuvant chemoradiation therapy

Patients with more advanced tumours are usually treated with a long course of CRT in order to facilitate tumour downstaging. Two multicenter randomised trials have demonstrated the benefit of additional chemotherapy to radiotherapy, leading to a higher complete response rate and a lower local recurrence rate after 5 years follow-up [6,7]. CRT can potentially increase the number of patients who undergo sphincter sparing surgery, although only few authors have demonstrated this in a prospective trial [8-10].

A pathological complete tumour response (pCR) following long-course CRT is reported in 8 - 27% of the patients [10-16]. Patients with a complete response have an improved overall survival and local recurrences in patients with ypT0 and ypT1 tumours are low (0% - 6%). The additional value of TME surgery in case of a pCR after CRT is questioning. The selection of patients with a pCR is difficult using imaging techniques and transanal excision of the remaining tissue or residual tumour is probably the most profound method.

### Local excision/TEM

Transanal local excision (LE) is performed in patients with benign and low-risk superficial malignant rectal neoplasms. Transanal endoscopic microsurgery (TEM) is a local excision technique which enables the surgeon to perform a full thickness excision with great precision. An operating rectoscope with a diameter of 4 cm and a length of 12 or 20 cm is used. The scope has four work channels, a stereo optic vision channel, a light source, and an insufflation port to obtain a pneumorectum for maximum exposure. TEM is superior to local excision according to Parks for benign and malignant rectal neoplasms. There is a significantly lower risk of irradical or incomplete resection and consequently a lower recurrence rate [17-19]. TEM is also considered an alternative for patients who are unsuitable for major surgery because of medical comorbidity or those patients who require an APR but refuse a colostomy. Unfortunately, local excision leads to higher rates of local recurrence and survival may be compromised compared to radical TME surgery except for low-risk T1 tumours [20,21].

### Lymph node involvement

The key to appropriate use of LE for rectal cancer is patient selection; accurate preoperative primary tumour staging and prediction of lymph node involvement. For detection of nodal disease, ERUS and MRI have a similar sensitivity (67% versus 66%) and specificity (78% versus 76%) [22]. However, both examinations are highly operator dependent. MRI provides excellent imaging of the rectum, mesorectum, fascia propria of the rectum, and other pelvic structures and is useful for determining the risk of CRM involvement preoperatively. These aspects are not relevant to early-stage patients considered for local excision. The risk of lymph node involvement increases with depth of invasion of the rectal wall. Even in patients with a T1 stage, there can be lymph node involvement. The incidence of lymph node metastasis ranges from 6% to 14% for T1 tumours, 17% to 23% for T2 tumours, and 49% to 66% for T3 tumours [23]. The percentage of patients with involved lymph nodes is much lower after neoadjuvant CRT [24,25], probably because CRT has sterilised tumour containing lymph nodes. Others have shown that there is a correlation between the T stage and the N stage after CRT [26-29].

This effect of neoadjuvant CRT enables a combined treatment modality with local excision.

## Methods/Design

### Hypothesis

Since 8% to27% of the patients receiving neoadjuvant CRT for locally advanced rectal cancer has a pCR [10-15], a rectum saving treatment should be achievable for a number of patients with distal rectal cancer. In the CARTS-study, patients will be treated with CRT aiming at reaching a (near) pCR. By excision of the primary tumour site by TEM patients with a pathological (near) pCR (ypT0-1) can be identified.

### Objectives

Primary objective of the study is to determine the number of patients with minimal residual disease (ypT0-1) after neoadjuvant CRT followed by TEM surgery. The resection specimen should be complete (> 2 mm margin) without evidence of nodal metastases (if nodes are found in the full thickness specimen). Secondary objectives are quality of life and local recurrence rate.

### Design

**Figure 1:**
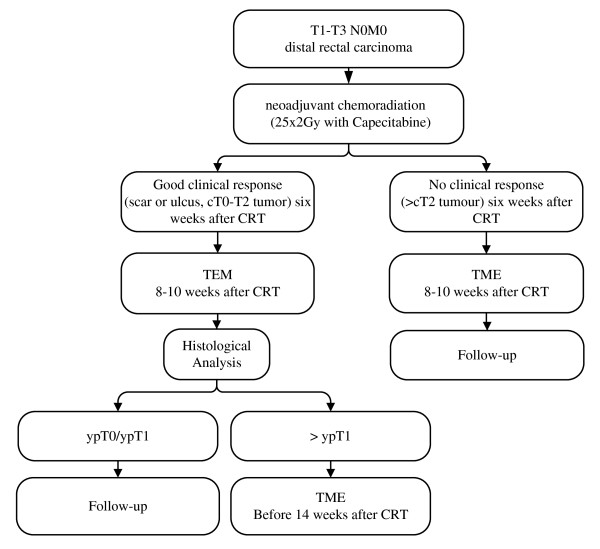
**flowchart of the CARTS-study**.

This study is a non-randomized feasibility study to determine whether radiotherapy combined with capecitabine followed by organ-sparing surgery using TEM can be considered as a valid new treatment modality in distal rectal cancer. The flow chart of the study is showed in Figure 1.

### Study population

Patients who meet the following inclusion criteria are eligible for participation in this trial: diagnosis of a distal adenocarcinoma within 10 cm of the anal verge, staged as cT1-T3 tumour on imaging, age > 18 years and written informed consent.

Exclusion criteria are: Low risk T1, tumour ineligible for TEM (circular or intra-anal tumour), pre-existing faecal incontinence (soiling is not), synchronous tumours, or presence of mesorectal lymph nodes larger than 5 mm on CT, MRI and/or endorectal ultrasound and contraindications for capecitabine.

### Participating centres

At least 15 Dutch hospitals will participate in this study, including 3 university medical centers.

### Interventions

#### Neoadjuvant chemoradiation therapy

All patients will receive neoadjuvant CRT, consisting of radiotherapy with a total dose of 50 Gy which is given in 25 fractions during 5 weeks. Patients will receive 825 mg/m^2 ^capecitabine b.i.d. 7 days per week during the whole treatment period. Six weeks after ending the CRT, therapy effect is evaluated by MRI, rectoscopy and ERUS. Patients with a clinical T0-2 tumour after CRT will undergo LE using TEM 8 - 10 weeks after the end of the neoadjuvant treatment. All other patients will undergo TME surgery.

After histological examination of the resected specimen, all patients with an ypT2-3 tumour, positive resection margins, or lymphangioinvasive growth, will undergo radical surgery within 4-6 weeks after the TEM-procedure.

#### TEM

TEM will be performed as described by Buess [30]. Under general/spinal anesthesia, a specialized TEM rectoscope of 12 or 20 cm in length (Wolf GmbH Knittlingen or Storz GmbH Tuttlingen, Germany) is inserted within the rectum to assure proper visualization of the lesion. The rectal cavity is insufflated with CO_2 _by a combined endosurgical unit to achieve constant distension for appropriate visualization of the rectal neoplasm. The combined endosurgical unit further regulates irrigation and suction, thereby maintaining a constant intrarectal pressure.

### Outcome parameters

#### Primary outcome

Primary objective of the study is to demonstrate that more than 12 of 55 patients have minimal residual disease 8 - 10 weeks after neoadjuvant CRT. Minimal residual disease is defined as an ypT0 or ypT1 stage.

#### Secondary outcome

Secondary objectives are local recurrence rate (LRR) and quality of life after the given treatment. All participating patients will have intensive follow-up during three years to evaluate the presence of locoregional and distal recurrent disease. Patients will be evaluated at the outpatient clinic every three months and MRI of the pelvis and CT scan of the thorax and abdomen are performed every six months. Patients who have been successfully treated with TEM will be followed with rectoscopy and ERUS every three months. Quality of life will be measured with the EORTC-QLQ-C30 and the EORTC-QLQ-C38 before CRT and four times during follow-up.

The following issues will also be registered and analysed for both the patients who undergo TEM and those undergoing TME: treatment related toxicity of the preoperative CRT and postoperative complications. The number of positive lymph nodes in TME patients will be assessed. This will give valuable information on the preoperative imaging modalities. All patients will be asked to consent with storage of tumour biopsies for translational research.

### Sample size calculation

Fifty-five patients will be included in this study. The study treatment protocol is considered successful if 30% or more of the included patients will complete CRT and undergo TEM surgery with complete resection of the ypT0-1 tumour. This means a resection with > 2 mm resection margin. A response of 15% or less will be considered a failure of this treatment modality. A three-step model for phase II cancer clinical trials will be used for calculating patient numbers with an alpha and beta of 0.1. An evaluation will be planned after 20 and 33 patients.

### Ethics

The medical ethical committee of the Radboud University Nijmegen Medical Centre has approved the study protocol (NL 2882.091.10). The CARTS study is registered at clinicaltrials.gov (NCT01273051)

Prior to registration written informed consent will be obtained in all patients.

## Discussion

During the last decade, the introduction of TME-surgery and neoadjuvant treatment strategies has led to an improved overall and disease free survival in patients with rectal cancer [31-34]. With this improvement of oncological outcome, the question has risen if new treatment modalities can be developed with less morbidity and mortality and an increase in quality of life.

Local excision via TEM of rectal tumours has demonstrated to be a technique with significantly lower morbidity and mortality rates and hospital stay compared to standard TME surgery [21,35]. Recently, Doornebosch et al. demonstrated that functional outcome was also better after TEM in comparison with TME. However, TEM as a solitary procedure is not considered an oncological save treatment. Even in T1tumours, local recurrences are reported in 6-18% of the patients [20]. For T2 or T3 carcinomas, local recurrence rates are unacceptable high [36-38] and LE is only performed in such patients if they are physically unfit to undergo standard rectal surgery [39]. Therefore, alternative strategies should be accounted for in order to improve the oncological results after LE (TEM). Postoperative radiotherapy or CRT has been used in several centers after TEM for rectal cancer, but did not lead to acceptable results [38,40]. Neoadjuvant treatment with CRT is another strategy which is already generally used in locally advanced rectal carcinomas with acceptable toxicity [10,41].

After CRT, pCR has been reported in 8-27% of the patients [10-15]. This has led to evaluation of a wait and see strategy for patients with a clinical complete response (cCR), which is currently investigated for oncological safety by several investigators [42]. However, a pathological evaluation of the tumour remnant has several potential advantages to select patients for rectum sparing treatment. First of all, cCR is not always a pCR [42-45]. We have described a patient with a cCR, which demonstrated to be an ypT2 tumour after TEM, but eventually turned out to be a ypT3N1 tumour after TME surgery [45]. In these patients who seem to have a favourable response, a wait and see policy will delay adequate mesorectal excision. Secondly, a clinical partial response can be a pCR. In order to prevent patients with palpable tumour scarring from undergoing TME surgery, a biopsy of the scar seems to be the best option. Thirdly, patients with a near complete response after CRT (ypT1) can be adequately treated with a full thickness excision of the rectal wall.

The combination of TEM and CRT has been studied retrospective [46] and is studied in a similar protocol in the USA (ACOSOG Z0641 study) [47]. Local recurrence after a pCR (ypT0) has never been described, and also the local recurrence rate after a near complete response (ypT1) is reported to be low (0-6%). For ypT2 carcinomas, varying LRR have been described but are likely to be above 10% [46]. Despite these observations the ACOSOG Z0641 protocol prescribes a wait and see policy for ypT2 tumours [48]. We feel that this might not be an oncological save treatment and have therefore chosen to perform TME surgery in all patients with ypT2 and higher tumours and monitor long term outcome. Another interesting observation will be the registration of complications after TEM. In the case reports described in the literature, several patients experienced wound complications, but the exact rate will be established in this prospective analysis [49].

The trial accrual is expected to be adequate in the above mentioned centers and it is assumed that in two years all patients will be treated. This trial will give us an answer if this multimodality protocol for rectum saving treatment of rectal cancer is feasible and if so, a randomized phase III trial will be conducted.

## Competing interests

The authors declare that they have no competing interests.

## Authors' contributions

GMJB, prepared the manuscript, coordinates the study and is the corresponding author. EJRG, is one of the principal investigators. CJAP, is one of the principal investigators. IDN, is one of the members of the writing committee. HR, is one of the members of the writing committee. JJMEN, is one of the principal investigators. EM, is one of the principal investigators. PGD, is one of the members of the writing committee. PJT, is one of the members of the writing committee. EJD, is one of the members of the writing committee. RSD, is one of the members of the writing committee. AC, is one of the members of the writing committee. CAMM, is one of the members of the writing committee. RAEMT, is one of the members of the writing committee. IHJTH, is one of the members of the writing committee. HJTR, is one of the members of the writing committee. GPS, is one of the members of the writing committee. AJT, is one of the members of the writing committee. JWAL, is one of the members of the writing committee. GL, is one of the members of the writing committee. GLB, is one of the members of the writing committee. TJA, is one of the members of the writing committee. AP, is one of the members of the writing committee. ERM, is one of the members of the writing committee. CH, is one of the members of the writing committee. AJAB, is one of the members of the writing committee. CV, is one of the members of the writing committee. JHWW, supervised the first author and is the initiator of the study

All authors read and approved the final manuscript

## Pre-publication history

The pre-publication history for this paper can be accessed here:

http://www.biomedcentral.com/1471-2482/11/34/prepub
